# Projecting Suitability and Climate Vulnerability of *Bhutanitis thaidina* (Blanchard) (Lepidoptera: Papilionidae) with Conservation Implications

**DOI:** 10.1038/s41598-019-51972-6

**Published:** 2019-10-28

**Authors:** Shao-Ji Hu, Dong-Hui Xing, Zhi-Xian Gong, Jin-Ming Hu

**Affiliations:** 1 Yunnan Key Laboratory of International Rivers and Transboundary Eco-security, Yunnan University, Kunming, 650500 China; 2 Institute of International Rivers and Eco-security, Yunnan University, Kunming, 650500 China; 3 School of Agriculture, Yunnan University, Kunming, 650500 China; 4 Yulong Xueshan Provincial Nature Reserve, Yulong, 674100 China

**Keywords:** Conservation biology, Entomology, Biodiversity

## Abstract

*Bhutanitis thaidina* is an endemic, rare, and protected swallowtail in China. Deforestation, habitat fragmentation, illegal commercialised capture, and exploitation of larval food plants are believed to be the four major causes of population decline of *B. thaidina* in the recent decade. However, little attention was paid to the impact of climate change. This study used ecological niche factor analysis and species distribution model to analyse the current suitable areas for *B. thaidina* with BioClim variables as well as its future suitable areas under four future climate scenarios (represented by four Representative Concentration Pathways: RCP2.6, RCP4.5, RCP6.0, and RCP8.5). Statistical analysis was carried out to compare the possible area and altitude changes to the distribution of *B. thaidina* under changing climate. Our analyses showed that the suitable areas for *B. thaidina* are fragmented under the current climate, with four suitable centres in northwestern Yunnan, northeastern Yunnan and northwestern Guizhou, the western margin of Sichuan Basin, and Qinling mountains. Apart from further habitat fragmentation under climate change, slight range expansion (average 6.0–8.9%) was detected under the RCP2.6 and RCP4.5 scenarios, while more range contraction (average 1.3–26.9%) was detected under the RCP6.0 and RCP8.5 scenarios, with the two southern suitable centres suffering most. Also, a tendency of contraction (2,500–3,500 m) and upslope shift (~600 m) in suitable altitude range were detected. The findings of this study supported the climate-vulnerable hypothesis of *B. thaidina*, especially under future climate like the RCP6.0 and RCP8.5 scenarios, in terms of contraction in suitable areas and altitude ranges. Conservation priority should be given to northwestern Yunnan, northeastern Yunnan, and northwestern Guizhou to alleviate the stress of massive habitat loss and extinction. Refugial areas should be established in all four suitable centres to maintain genetic diversity of *B. thaidina* in China.

## Introduction

**Figure 1:**
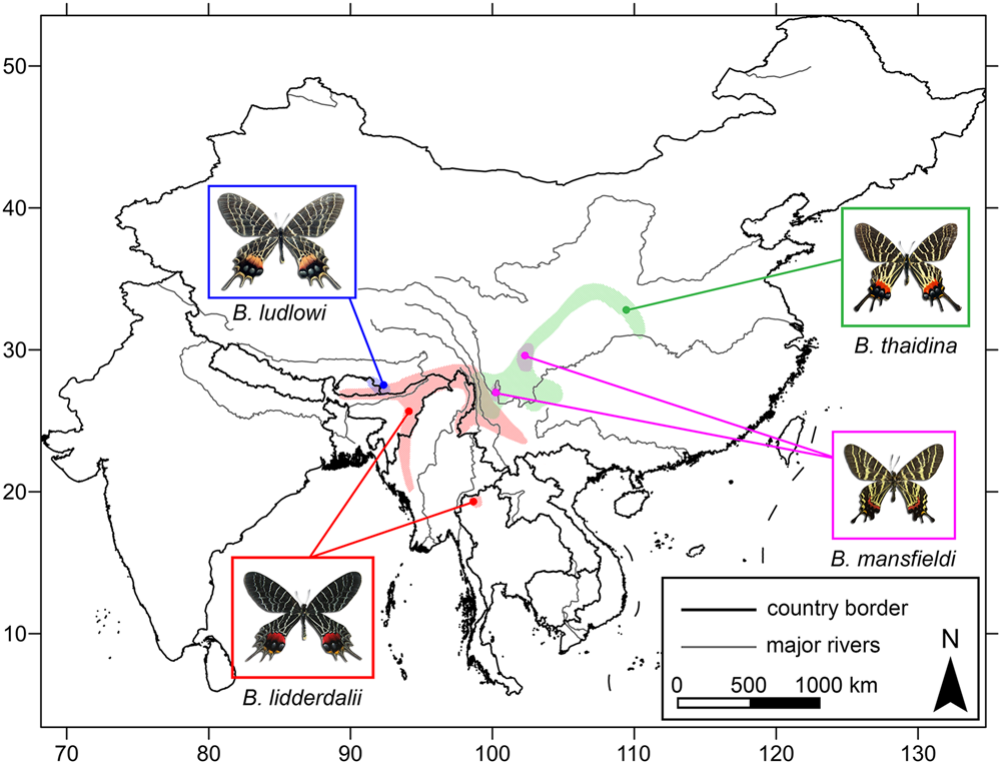
Tentative distribution range of four known species of *Bhutanitis* swallowtails. Photo of *B. ludlowi* © The Research Institute of Evolutionary Biology, Tokyo, Japan; photos of *B. lidderdalii*, *B. thaidina*, and *B. mansfieldi* © Shao-Ji Hu.

*Bhutanitis* (Atkinson) (Lepidoptera: Papilionidae: Parnassiinae) is a group of world-class rare and regional endemic swallowtails (CITES Appendix II, ICUN enlisted)^1,2^ comprising only four known Sino-Himalayan species (Fig. 1), namely *B. ludlowi* Gabriel, *B. lidderdalii* (Atkinson), *B. thaidina* (Blanchard), and *B. mansfieldi* (Riley)^3–11^. China, especially its southwest part, is the diversity centre of these butterflies, containing the last three species^10,11^.

*Bhutanitis thaidina* and *B. mansfieldi* are two National Grade II protected species of high conservation value endemic to southwest China^12^. In the past three decades, population decline has been observed in both species mainly due to deprivation of habitat linked to human activities. Deforestation for firewood, habitat fragmentation by agriculture and infrastructure expansion; illegal commercialised capturing for overseas markets; and exploitation of larval food plants (*Aristolochia* spp.) for traditional herbal medicines were believed to be the main aspects^4,13–15^.

Habitat losses associated with human activities are undoubtedly imminent threats to the survival of certain populations of these two *Bhutanitis* species in China. In recent years, a few conservation studies were carried out on *B. thaidina* in attempt to alleviate the situation from biological and ecological aspects^4,14,15^, while little could be done with *B. mansfieldi*, a bionomic and distribution data-poor species. Apart from human activities, climate change is another factor which is attributed to many cases of extinction of rare and endemic species globally^16,17^. However, unfortunately, little attention has been paid to such slow but prolonged effect of climate change on the future of these butterflies.

Species distribution models (SDM) contains a range of effective analytical tools for simulating and visualising suitable areas (potential distribution range) of organisms, and has been widely applied to species of conservation interests as well as policy making over the past decade^18–29^. Among these methods, ecological niche factor analysis (ENFA) and maximum entropy (MaxEnt) modelling are the most frequently applied SDMs which project the suitable area of a species using the presence-only data without depending on bionomical parameters of the focal species, or being biased by pseudoabsence data^30,31^.

In an attempt to fill the gap in conservation of *Bhutanitis*, the present study chose *B. thaidina*, a data-rich species as our model, analysed the current distribution and the future distribution shift under different climatic change scenarios^32^ using SDMs of ENFA and MaxEnt. The results will provide us an overview of its suitable areas in China and facilitate our understanding of how the suitable areas would shift in the process of climate change. The findings of the present study are beneficial to conservation management in current time as well as to formulate countermeasures to alleviate population decline of this rare butterfly in the future.

## Materials and Methods

### Data sources

**Figure 2:**
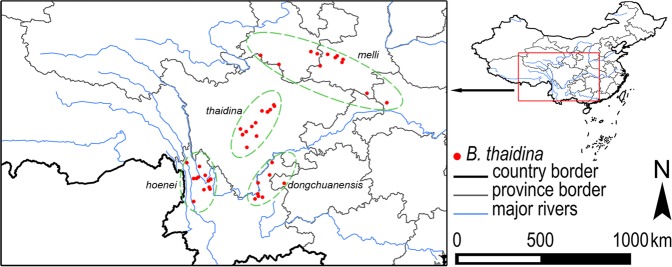
Distribution of the presence data points of *B. thaidina* in China with designation of subspecies range.

Species distribution points were extracted from specimen collections (Natural History Museum, London; Zoologisches Forschungsinstitute und Museum Alexander König, Bonn; Institute of Zoology, Chinese Academy of Sciences (CAS); Kunming Institute of Zoology, CAS; Southwest Forestry University; and private collections), literature^3,4,9,14,33–37^, and web databases and photo records (www.papc.cn; www.flickr.com) (Table S1). In total, 61 distribution points for *B. thaidina* were obtained (Fig. 2). All coordinates were transformed to decimal degrees and stored in an Excel spreadsheet for further use.

Nineteen BioClim^38^ variables were used to represent the current climate features (averaged over 1970–2000), the 19 BioClim variables and the altitude mask with 30 arc seconds resolution were obtained from the WorldClim database (www.worldclim.org). All data was further cropped by the political boundary of the People’s Republic of China, and will be referred as ‘environmental factors’ hereafter.

The CMIP5 climate projections under the IPCC-AR5 (the 5^th^ Assessment Report of the Intergovernmental Panel on Climate Change) frame were used to represent the future climate^32^. Four representative concentration pathways (RCPs), RCP2.6, RCP4.5, RCP6.0, and RCP8.5 were selected to simulate possible climate changes^32^. Data with 30 arc seconds resolution was also obtained from the WorldClim database and cropped by the political boundary of the People’s Republic of China.

Species distribution points and environmental factors were transformed into two formats, with the IDRISI format for ENFA analysis^31,39^ and the ASCII format for MaxEnt analysis^40^.

### Frequency and importance of environmental factors

Ecological niche factor analysis (ENFA) was performed in Biomapper 4.0^31^ for current climate only, as future models cannot be inferred from current distribution data. To minimise possible negative influence of autocorrelation between environmental factors, correlation of the 20 environmental factors were tested using a UPGMA dendrogram in Biomapper 4.0, and factors with correlation coefficients above 0.95 were removed from the dataset. When removing autocorrelated factors, those representing short-period extremes (e.g., minimum temperature of the coldest month, maximum precipitation of the wettest month) were removed, while those representing longer periods (e.g., mean temperature of the coldest quarter, precipitation of the driest quarter) were kept, as such type of environmental factors often play an important role in species distribution.

In an attempt to analyse the distribution prevalence of *B. thaidina*, values of previously screened environmental factors in the distribution area of *B. thaidina* and the entirety of China were extracted using DIVA-GIS 5.7 (www.dive-gis.org)^41^. The distribution frequencies were calculated in Biomapper 4.0. The importance of the environmental factors was measured using the jackknife method in MaxEnt 3.4.1 with 1,000 iterations^42,43^.

### Species distribution model (SDM)

Environmental factors with ENFA scores over 0.2 were selected and assigned to the MaxEnt 3.4.1^40^ to project suitable areas (current and future climate variables were analysed separately) based on the presence data points, among which 25% were extracted for random testing. The logistic output method was selected to estimate the distribution (or presence) probability of *B. thaidina* considering certain assumptions of species’ prevalence and sampling effort^44^. The resultant map was saved as ASCII format and then redrawn using Surfer 10.0 (Golden Software Inc., Golden, CO, USA). Model robustness was evaluated using the receiver operation curve (ROC) and the area under the ROC curve (AUC)^45,46^, where the AUC value [AUC ∈ (0, 1)] approaching 1.0 is usually considered acceptable, whereas it should be rejected when approaching the random turquoise line of 0.5^47^.

### Statistical analyses

The number of grid cells (further transformed into area using 1 grid cell = 1 km^2^) as well as their elevation property were extracted in ArcGIS 10 (ESRI, USA) from projection maps under the current climate and the four future climate scenarios in both the 2050 s and the 2070 s. Comparative bar charts for suitable areas and curve line charts for suitable altitude range were made to 2050 s vs. current, 2070 s vs. current, and 2070 s vs. 2050 s, mainly focusing on suitability ranks from 0.5 to over 0.8.

## Results

### Key environmental factors

Nine environmental factors, Alt, Bio5, Bio6, Bio8, Bio9, Bio13, Bio14, Bio18, and Bio19, were removed from the dataset due to strong autocorrelation (correlation coefficients >0.95) in UPGMA dendrogram test. ENFA analysis using the remaining eleven environmental factors further excluded Bio15 and Bio17 as all scores of ecological factors were under 0.2 (Table 1). The final remaining nine environmental factors, Bio1, Bio2, Bio3, Bio4, Bio7, Bio10, Bio11, Bio12, and Bio16, were key influential factors of the current suitability for distribution of *B. thaidina* in China (Table 1).

**Table 1:** Score matrix of current key environmental factors screened by ENFA analysis.

EFs	F1	F2	F3	F4	F5	F6	F7	F8
	62%	24%	5%	5%	2%	1%	1%	1%
Bio1	0.16	0.62	−0.52	0.09	0.21	0.06	−0.04	0.47
Bio2	−0.28	−0.10	−0.23	0.28	0.12	0.04	0.07	0.02
Bio3	0.38	0.08	0.11	−0.10	−0.05	−0.03	−0.01	−0.01
Bio4	−0.47	0.41	−0.13	0.12	−0.35	0.56	−0.42	−0.35
Bio7	−0.48	0.09	0.47	−0.71	−0.25	−0.07	−0.17	−0.08
Bio10	−0.06	−0.64	0.13	0.27	0.39	−0.53	0.54	0.14
Bio11	0.32	0.05	0.49	−0.55	−0.78	0.63	−0.70	−0.80
Bio12	0.28	0.06	0.30	−0.07	0.01	0.01	−0.04	0.00
Bio15	−0.07	−0.04	0.13	−0.05	0.00	0.00	−0.01	0.01
Bio16	0.33	0.00	−0.24	0.06	0.01	0.01	0.05	−0.02
Bio17	−0.02	−0.09	−0.04	−0.04	−0.01	−0.01	0.02	0.02

EF = environmental factors, F = ecological niche factors identified by ENFA analysis. Marginality = 1.43, speciality = 7.12, tolerance = 0.14.

Frequency distribution of *B. thaidina* against the nine key influential environmental factors for the entirety of China showed evident preference for each factor. For temperature factors, *B. thaidina* occurs in areas where annual mean temperature (Bio1) ranges between 1–17 °C, mean temperature of the warmest quarter (Bio10) ranges between 10–23 °C, and mean temperature of the coldest quarter (Bio11) ranges between −9–10 °C (Fig. S1). For precipitation factors, *B. thaidina* occurs in areas where annual precipitation (Bio12) ranges between 630–1,400 mm and precipitation of the wettest quarter (Bio16) ranges between 250–750 mm (Fig. S1). For temperature variabilities, *B. thaidina* mainly occurs in areas where mean diurnal temperature range (Bio2) varies between 7–12 °C, temperature annual range (Bio7) varies between 22–35 °C, relatively higher isothermality (Bio3) and low temperature seasonality (Bio4) (Fig. S1).

### Current suitable areas

The MaxEnt analysis produced a projection with the training AUC = 0.983 and the testing AUC = 0.981, representing a credible result of the suitability distribution for *B. thaidina* under the current climate.

**Figure 3:**
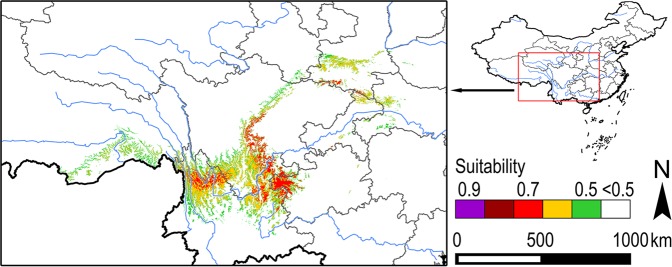
Suitable areas for *B. thaidina* in China under current climate condition.

On the large scale, the current suitable areas for *B. thaidina* are still confined to west China, as mirrored by its actual distribution localities (Fig. 2). Four areas with higher suitability were identified. (1) Northwest Yunnan. This area occupies the Hengduan Mountains in Yunnan and southwest Sichuan, including the mountains separated by the upper Irrawaddy, Salween, Mekong, and Yangtze watersheds. The eastern edge of this area approximately reaches Anning River (a branch of upper Yangtze River), while the southern edge of it reaches east Dali to Yunlong (Fig. 3). (2) Northeast Yunnan, northwest Guizhou, and the western edge of Sichuan Basin. A larger stripe-shaped area initiates from Dongchuan and Luquan of east Yunnan altiplano and the west part of Bijie area in northwest Guizhou, runs northward to the north of Ya’an, and then turns northeastwardly to the border of south Gansu, with a gradient reduction of suitability (Fig. 3). (3) Taibai Shan and Daba Shan areas in south Shaanxi. Two small patches are separated by the Qinling ridge, reaching the southern edge of Hanzhong Plain in the north, and the northern edge of Sichuan Basin in the south (Fig. 3). (4) East Daba Shan and Shennongjia areas in the juncture of northeast Chongqing and northwest Hubei. A smaller patch with relatively lower suitability (Fig. 3).

At smaller and local scales, the suitable areas for *B. thaidina* are highly fragmented, even within the four isolated patches mentioned above. Despite ridges of high mountains and deep valleys of large rivers cutting these patches into separate pieces, the suitable areas for *B. thaidina* are further isolated by complex terrains in a small range (Fig. 3).

### Future change of suitable range

The MaxEnt analysis produced a projection with the training AUC = 0.983–0.986 and the testing AUC = 0.986–0.987, representing credible results of the suitability distributions for *B. thaidina* under the four future climate scenarios (four RCPs) in the 2050 s and the 2070 s, respectively.

**Figure 4:**
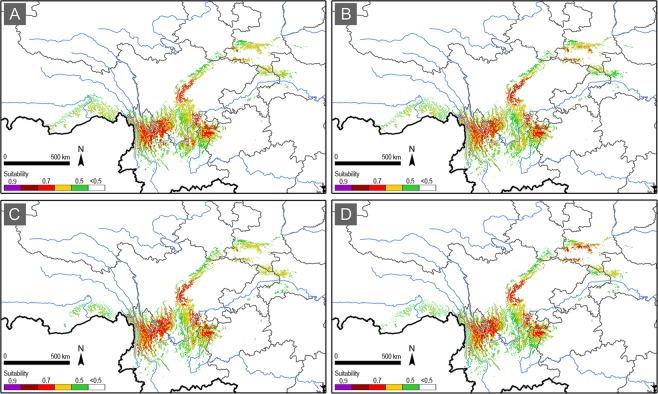
The suitable areas for *B. thaidina* in China in the 2050 s under the RCP2.6 (**A**), RCP4.5 (**B**), RCP6.0 (**C**), and RCP8.5 (**D**) scenarios.

In the 2050 s, the overall distribution pattern of the suitable areas showed obvious but non-radical changes. The change under RCP2.6 scenario is very limited, making the distribution pattern very similar to that under the current climate, but the 0.7 grade suitable areas expanded in northwest Yunnan, occupying the 0.6 grade suitable areas under the current climate; while the 0.7 grade suitable areas retreated in southwest Sichuan bordering with northeast Yunnan (Fig. 4A). Under the RCP4.5 scenario, the distribution pattern remained the same with that under the RCP2.6 scenario, except for an elevation of suitability grade (0.8–0.9) in the western edge of the Sichuan basin (Fig. 4B). Under the RCP6.0 and RCP8.5 scenarios, distribution pattern changed more obviously with the low-medium suitability grades (0.5–0.6) retreating in the southern edge and lower-altitude areas of the distribution range, but a higher suitability grade (0.8–0.9) appearing in northwest Yunnan and the western edge of the Sichuan basin (Fig. 4C,D). Gain of high suitability grade (0.7) was also detected in south Qinling under the RCP8.5 scenario (Fig. 4D).

**Figure 5:**
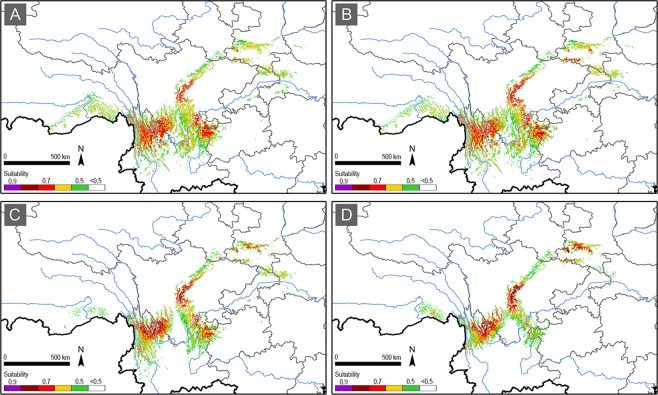
The suitable areas for *B. thaidina* in China in the 2070 s under the RCP2.6 (**A**), RCP4.5 (**B**), RCP6.0 (**C**), and RCP8.5 (**D**) scenarios.

In the 2070 s, the overall distribution pattern of the suitable areas under the RCP2.6 and the RCP4.5 scenarios almost remained the same as that in the 2050 s (Fig. 5A,B). However, dramatic changes to the suitability distribution under the RCP6.0 and the RCP8.5 scenarios were detected. Not only the low-medium suitability grades (0.5–0.6) largely retreated in the southern margin and the lower-altitude areas, the medium-high suitability grades (0.6–0.8) also dramatically retreated in the southern portion of the distribution range, especially in northwest Yunnan, northeast Yunnan, and northwest Guizhou (Fig. 5C,D). Similar to the 2050 s, gain of higher suitability grade (0.8–0.9) was detected in south Qinling under the RCP8.5 scenario (Fig. 5D).

### Future change of suitable area and altitudes

**Figure 6:**
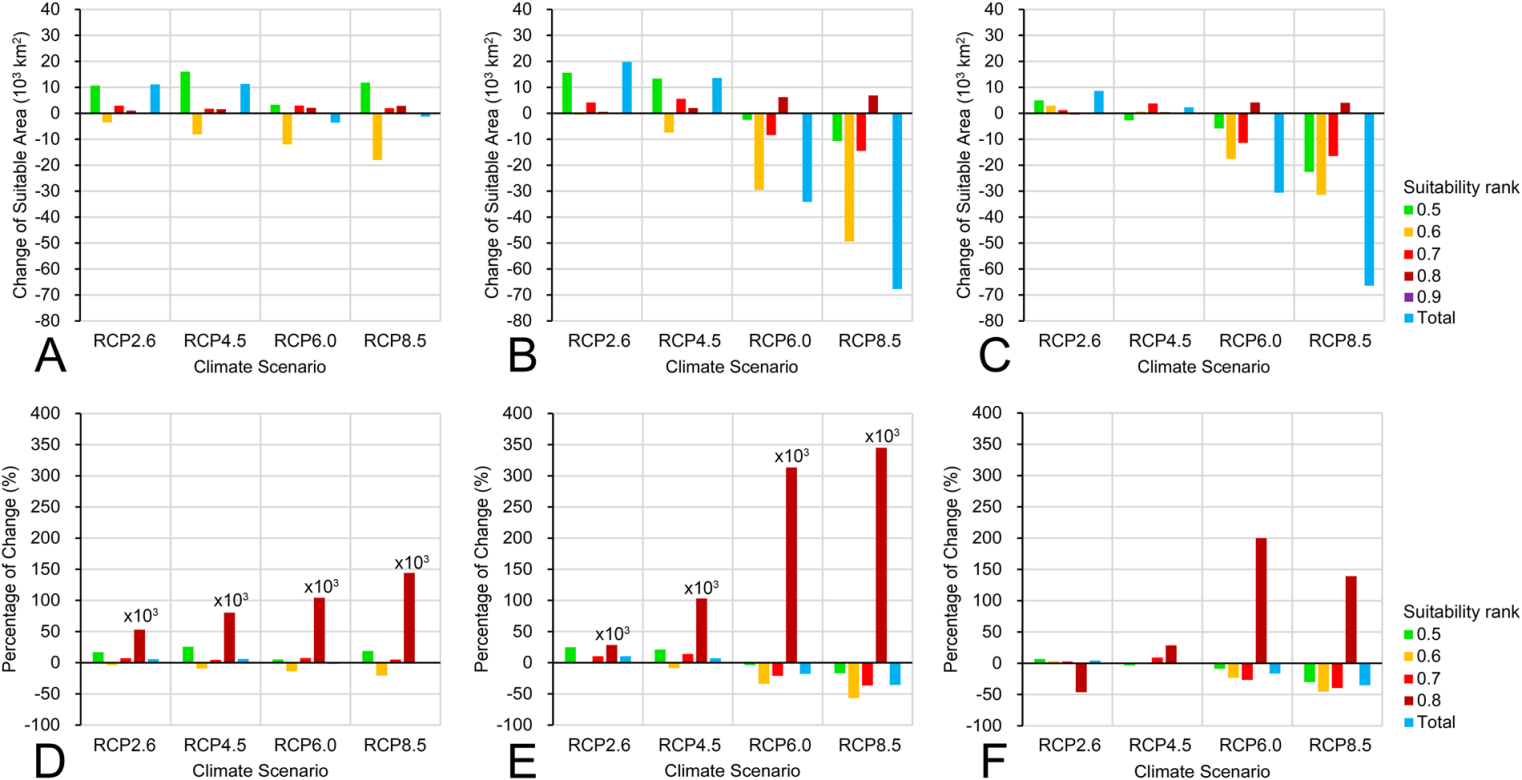
The change of suitable areas (10^3^ km^2^) (**A**–**C**) and the percentage of suitable area change (%) (**D**–**F**) for *B. thaidina* in China: (**A,D**) 2050 s vs. current, (**B,E**) 2070 s vs. current, (**C,F**) 2070 s vs. 2050 s.

Our quantitative analysis of the change of suitable areas showed that, in the 2050 s, suitable areas of the 0.5–0.6 and 0.7–0.9 ranks all increased under all future climate scenarios, with the 0.5–0.6 rank increased most (3.32–16.07 × 10^3^ km^2^, average 16.6%) and the 0.8–0.9 rank increased least (1.06–2.88 × 10^3^ km^2^, average 95,487.5%); however, suitable areas of 0.6–0.7 rank decreased significantly under all future climate scenarios (3.51–17.98 × 10^3^ km^2^, average 12.0%) (Fig. 6A,D). The total suitable areas for *B. thaidina* increased 11.14 × 10^3^ km^2^ (5.9%) and 11.30 × 10^3^ km^2^ (6.0%) under RCP2.6 and RCP4.5 scenarios, while they decreased 3.55 × 10^3^ km^2^ (1.9%) and 1.27 × 10^3^ km^2^ (0.7%) under RCP6.0 and RCP 8.5 scenarios (Fig. 6A,D).

In the 2070 s, suitable areas of the 0.5–0.6 and 0.7–0.8 ranks increased under RCP2.6 and RCP4.5 scenarios (13.39–15.64 × 10^3^ km^2^ and 4.17–5.59 × 10^3^ km^2^ respectively; average 23.1% and 12.3% respectively) but decreased under RCP6.0 and RCP8.5 scenarios (3.96–17.04 × 10^3^ km^2^ and 21.11–36.49 × 10^3^ km^2^ respectively; average 10.5% and 28.8% respectively), the suitable areas of the 0.8–0.9 rank increased under all future climate scenarios (0.57–6.90 × 10^3^ km^2^, average 197,500.0%); however, suitable areas of 0.6–0.7 rank decreased significantly under all future climate scenarios (0.58–49.37 × 10^3^ km^2^, average 25.0%) (Fig. 6B,E). The total suitable areas for *B. thaidina* increased 19.80 × 10^3^ km^2^ (10.5%) and 13.63 × 10^3^ km^2^ (7.2%) under RCP2.6 and RCP4.5 scenarios, while they decreased 34.12 × 10^3^ km^2^ (18.0%) and 67.67 × 10^3^ km^2^ (35.7%) under RCP6.0 and RCP 8.5 scenarios (Fig. 6B,E).

Comparison between the 2070 s and the 2050 s showed a similar tendency, the total suitable areas for *B. thaidina* increased 8.66 × 10^3^ km^2^ (4.3%) and 2.33 × 10^3^ km^2^ (1.2%) under RCP2.6 and RCP4.5 scenarios, while they decreased 30.57 × 10^3^ km^2^ (16.4%) and 66.40 × 10^3^ km^2^ (35.3%) under RCP6.0 and RCP 8.5 scenarios (Fig. 6C,F).

**Figure 7:**
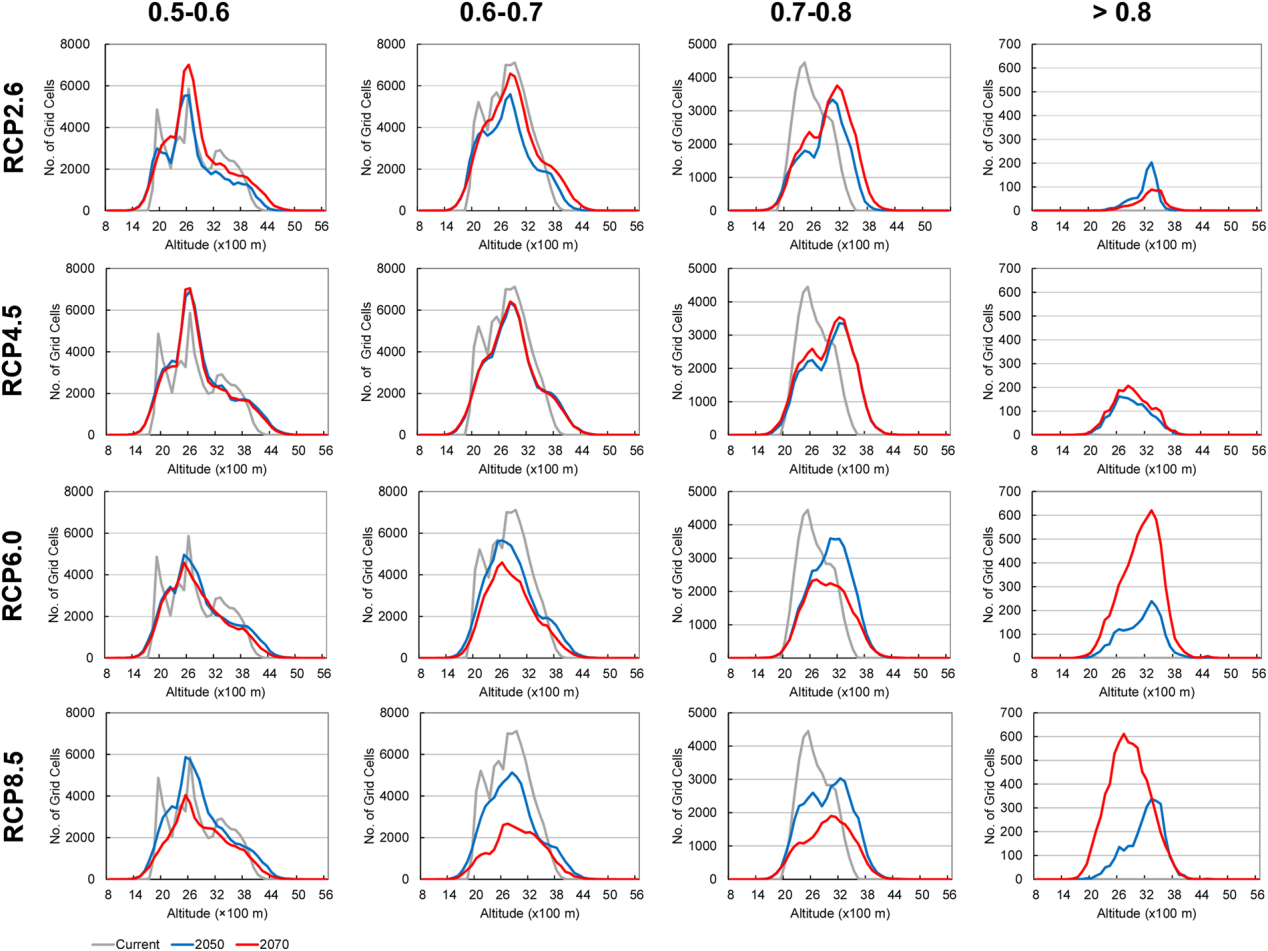
The frequency distribution of suitable altitudes for *B. thaidina* in China under different suitability ranks (0.5–0.6, 0.6–0.7, 0.7–0.8, >0.8) and the change under four future climate scenarios (RCP2.6, RCP4.5, RCP6.0, RCP8.5) in the 2050 s and 2070 s.

Analysis of frequency distribution change of suitable altitudes showed that, under RCP2.6 and RCP4.5 scenarios, the frequency distribution of suitable altitudes did not shift obviously but were more contracted between 2,500–3,200 m in the 0.5–0.6 and 0.6–0.7 ranks, while they shifted to 2,600–3,500 m in the 0.7–0.8 rank (Fig. 7). The frequency of suitable altitudes in the 2050 s is almost equal to that in the current, while the frequency of suitable altitudes in the 2070 s was significantly higher than that in the 2050 s (Fig. 7). Under RCP6.0 scenario, the frequency distribution patterns were similar to those under the preceding scenarios, but the peak frequency of the suitable altitudes was lower, with that in the 2070 s even significantly lower than the 2050 s in the 0.6–0.7 and the 0.7–0.8 ranks (Fig. 7). It is noticeable that under the RCP8.5 scenario, the peak frequency of the suitable altitudes was further significantly lowered, especially in the 2070 s (Fig. 7).

## Discussion

### Distribution shift and climate vulnerability

Based on the EGV frequency distribution characters, *B. thaidina* mainly occurs in temperate climate zones with less precipitation, relatively higher diurnal temperature range, and lower temperature seasonality (Fig. S1). Such habitat is represented by montane broadleaf forest and subalpine evergreen needle leaf forest, which may extend from 2,000 m to below the treeline in west China, with its lower and upper limits varying from the south to the north^48,49^. With climate change, the habitat belt will be forced to ‘move’ upslope^50^. However, shift rates of the lower and upper limits are not expected to be the same, and such asymmetric shift rates will eventually result in a decline in suitable altitude belt^51^.

Although some research indicates that species will move or expand their ranges upslope and poleward with climate change^52–56^, our analysis implied that *B. thaidina* would more likely suffer from rapid habitat compression or be driven to extinction during this process^57–59^. On the one hand, the distribution shift rate of species can hardly keep up with the pace of habitat shift and compression^51^; and on the other hand, ascent of the upper part of its suitable range will be limited by unfavourable climate or soil condition (e.g., increased precipitation, erosion linked to permafrost degeneration)^60^. More practically, the ability of *B. thaidina* to move from one habitat to another is largely limited by lack of connecting mountain ridges with suitable habitat, since its suitable area is already highly fragmented even under the current climate. Our speculation on this issue was similarly demonstrated in the tropical forests^61^.

The adult is the only stage at which all butterflies can achieve distant movement, while other stages from egg through pupa can only stay in the same locality. Such a life history further reduces their ability to escape from bad climate. *Bhutanitis*, a univoltine group with a maximum adult stage of only 2.5 months per year^14,62^, can hardly defend themselves against any climate induced incidents, including extreme precipitation, long-lasting drought, or even forest fire^63,64^. The extinction of *B. lidderdalii* on Doi Chiang Dao of northern Thailand in 1983, caused by a severe forest fire in the dry season as a result of the 1982–1983 El Niño, is a most recent case (A. M. Cotton, pers. comm.).

As a result, the distribution shift for *B. thaidina* in light of climate change would compress its suitable habitat and further reduce its refugial areas (Figs 3–7). Globally, the climate vulnerability of *B. thaidina* is much higher under the RCP6.0 and RCP8.5 scenarios compared to that under the RCP2.6 and RCP4.5 scenarios. Regionally, climate vulnerability of the two southern distribution centres is higher than the two northern ones (Figs 4 and 5) (discussed in detail below).

### Biodiversity significance

The current suitable areas revealed a patchy and highly fragmented distribution pattern for *B. thaidina* (Fig. 3), while future projection showed a compressed and further fragmented distribution pattern (Figs 4 and 5). Nonetheless, as the present study only applied climatic factors in SDM simulation, the actual distribution pattern of *B. thaidina* could be more fragmented on an unsuitable matrix when availability of host resources and vegetation are taken into consideration. In population genetics, highly fragmented distribution would result in a reduction of gene flow and genetic diversity^65–71^.

*B. thaidina* is a morphologically variable species with four subspecies recognised to date: ssp. *thaidina* in west Sichuan, ssp. *hoenei* Bryk in northwest Yunnan, ssp. *melli* Bryk in Qinling and Taibai Shan (probably also in Shennongjia), and ssp. *dongchuanensis* Lee in northeast Yunnan and northwest Guizhou^3,9,11,33,35,72^ (Fig. 2), mirroring our identification of four suitability centres (Figs 3–5). The biological and ecological issue underpinning the taxonomic complexity is that each subspecies possesses a distinct genetic profile. The genetic and morphological profiles altogether constitute the entire biodiversity integrity of *B. thaidina* in China, and any degeneration or loss of such profiles will directly lead to a loss of biodiversity of this endemic species.

The distribution pattern of suitable areas for *B. thaidina* is highly fragmented, thus making each subspecies a metapopulation comprised of multiple scattered and isolated smaller local populations. A recent population genetic analysis suggested very low genetic diversity among all populations of *B. thaidina* in China^73^, implying vulnerability to degeneration or extinction in the dynamic wild^74^. In the process of climate change, the future distribution pattern of *B. thaidina* will be further fragmented and isolated, which would inevitably bring more restriction to the gene flow between the four distribution centres as well as within each one. Our future projections showed significant suitability loss in the distribution range of ssp. *dongchuanensis* in northeast Yunnan and northwest Guizhou, followed by ssp. *hoenei* in northwest Yunnan (Figs 4 and 5), making these two subspecies more prone to extinction under the RCP6.0 and RCP8.5 scenarios, compared to the other two subspecies.

### Conservation implications

The present study showed that the fragmented suitable areas for *B. thaidina* will undergo further fragmentation and reduction in the process of climate change (Figs 3–5). Hence, maintaining current existing suitable areas is vital to the conservation of this rare species.

To conserve *B. thaidina* with genetic integrity, conservation strategies must firstly take all four suitable centres into equal consideration, as each suitable centre represents a distinct subspecies of *B. thaidina* in China. Next, combining the degree of rarity and vulnerability, priority should be given to areas with ssp. *dongchuanensis* and ssp. *hoenei*, which will be the most threatened in the future (Figs 4 and 5); followed by ssp. *melli*, which is only found in a narrow area in Qinling (Fig. 3); and ssp. *thaidina* being of least concern.

Availability of larval food plants is also crucial in conservation of *B. thaidina*. This species shows a strong host association in nature, and subspecies use different *Aristolochia* species, e.g., in northwest Yunnan and most parts of west Sichuan, *B. thaidina* mainly uses *A. moupinensis* and *A. delavayi*^3,14^, while using *A. mandshuriensis* in the Qinling Mountain area^4^. These food plant species have being exploited for traditional Chinese herbal medicines until recent years^75^. Such long lasting exploitation has already depleted wild resources of *Aristolochia* in some places^2^. Apart from human exploitation, deforestation of virgin forests on the median altitude to subalpine mountains is another important threat to wild *Aristolochia* resources, as most *Aristolochia* species are shade plants. Deforestation will also destroy the suitable habitats of *Aristolochia*.

Establishing refugial areas for *B. thaidina* could be an optimal *in situ* protection method. When selecting sites for refugial areas, vegetation surveys must be performed in advance to ensure the best vegetation type being included, e.g., *Quercus* stands associated with multiple local *Aristolochia* species^76^. However, the optimal planning of refugial areas for *B. thaidina* must rely on future in-depth research involving bionomics, dispersal capability, food plant adaptability, habitat matrix composition and connectivity, as well as a thorough evaluation of population genetic diversity.

By establishing refugial areas in such vegetation types, not only could *B. thaidina* be well conserved, but also many other rare, regional endemic, or data-poor butterflies can be protected under the umbrella-species effect^77,78^, e.g., *Bhutanitis lidderdalii*, *B. mansfieldi*, *Byasa daemonius* (Alphéraky), *B. plutonius* (Oberthür), *B. rhadinus* (Jordan) associated with *Aristolochia*^62^; as well as numerous Theclini hairstreaks (Lycaenidae) associated with *Quercus*^79^.

When *in situ* protection faces the challenge of high and progressive habitat fragmentation due to climate change found in the present study, coupled with the limited genetic diversity described earlier^73^, other measures must also be considered in the future to increase the genetic diversity and evolution flexibility of *B. thaidina* to respond to rapid environmental changes in a certain area. Possible measures include introducing *ex situ* populations containing new genetic profiles from other distribution areas, or even releasing laboratory genetic recombinants^80^.

## Supplementary information

Supplemantary information
