# Supplementary material for: Projecting Suitability and Climate Vulnerability of Bhutanitis thaidina (Blanchard) (Lepidoptera: Papilionidae) with Conservation Implications
Source: Sci Rep. 2019 Oct 28;9:15384. doi: 10.1038/s41598-019-51972-6 (PMC6817869; doi:10.1038/s41598-019-51972-6)
Supplement: Supplementary file 1 — Supplemantary information [file 41598_2019_51972_MOESM1_ESM.pdf]

Supplementary information

Projecting Suitability and Climate Vulnerability of *Bhutanitis thaidina* (Blanchard) (Lepidoptera: Papilionidae) with Conservation Implications

Shao-Ji Hu[1, 2, +], Dong-Hui Xing[3, +], Zhi-Xian Gong[4], Jin-Ming Hu[1, 2, *]

1. Yunnan Key Laboratory of International Rivers and Transboundary Eco-security, Yunnan University, Kunming, 650500, China
2. Institute of International Rivers and Eco-security, Yunnan University, Kunming, 650500, China
3. School of Agriculture, Yunnan University, Kunming, 650500, China
4. Yulong Xueshan Provincial Nature Reserve, Yulong, 674100, China

+ Joint first authors

* Corresponding author: hujm@ynu.edu.cn

TABLE S1 Presence-only data records used for ENFA and MaxEnt analyses in the present research.

Locality	Species	Lon.	Lat.	Value
Dalongtan, Shennongjia, Hubei	B_thaidina	110.311541	31.496622	1
Dalongtan, Shennongjia, Hubei	B_thaidina	110.306310	31.494298	1
Changsong Ping, Lijiang, Yunnan	B_thaidina	100.131330	27.043526	1
Forest Park, Dongchuan, Yunnan	B_thaidina	103.220353	26.113961	1
Lanni Ping, Dongchuan, Yunnan	B_thaidina	102.985180	26.170230	1
Luoxue, Dongchuan, Yunnan	B_thaidina	102.950082	26.298928	1
Jiaozi Xueshan, Luquan, Yunnan	B_thaidina	102.792020	26.017949	1
Heqing, , Yunnan	B_thaidina	100.226138	26.577384	1
Fangniu Ping, Heqing, Yunnan	B_thaidina	100.181021	26.684531	1
Jianchuan, Yunnan	B_thaidina	99.877868	26.545135	1
Tianchi, Yunlong, Yunnan	B_thaidina	99.293331	25.870900	1
Ludian, Lijiang, Yunnan	B_thaidina	99.454900	27.184844	1
Wagai, Judian, Lijiang, Yunnan	B_thaidina	99.567004	27.258471	1
Chongjiang River, Zhongdian, Yunnan	B_thaidina	99.953225	27.362652	1
Tuguancun, Zhongdian, Yunnan	B_thaidina	99.969232	27.366396	1
Yunshan Ping, Yulong Xueshan, Yunnan	B_thaidina	100.233790	27.129949	1
Baishui, Yulong Xueshan, Yunnan	B_thaidina	100.266980	27.135189	1
Heishui, Yulong Xueshan, Yunnan	B_thaidina	100.252892	27.145871	1
Napa, Deqen, Yunnan	B_thaidina	99.622975	27.867600	1
Cizhong, Deqen, Yunnan	B_thaidina	98.903219	28.075132	1
Tse-Kou (Yanmen), Deqen, Yunnan	B_thaidina	98.903249	28.078321	1
Weixi, Yunnan	B_thaidina	99.286562	27.175044	1
10 km SE of Weixi, Yunnan	B_thaidina	99.339444	27.766666	1
Xiao Weixi, Weixi, Yunnan	B_thaidina	99.062346	27.444749	1
Zhaotong, Yunnan	B_thaidina	103.624228	27.381016	1
Yanjin, Yunnan	B_thaidina	103.767829	28.092838	1
Qiaojia, Yunnan	B_thaidina	102.968013	26.930059	1
Weining, Guizhou	B_thaidina	104.456645	26.908322	1
Moupin, Sichuan	B_thaidina	102.827286	30.352020	1
Siao-Lou, Sichuan	B_thaidina	102.610339	30.138339	1
Lizi Ping, Shimian, Sichuan	B_thaidina	102.328858	28.952355	1
Yanzi Gou, Luding, Sichuan	B_thaidina	102.082319	29.687156	1
Xinxing, Luding, Sichuan	B_thaidina	102.079691	29.674463	1
Moxi, Luding, Sichuan	B_thaidina	102.138731	29.677025	1
Wolong, Sichuan	B_thaidina	103.187620	31.031050	1
Xuankou, Guanxian (Dujiangyan), Sichuan	B_thaidina	103.502852	30.964680	1
Erlang Shan, Sichuan	B_thaidina	102.288122	29.849450	1
Gudao, Erlang Shan, Sichuan	B_thaidina	102.235734	29.834715	1
Gongga Shan, Sichuan	B_thaidina	102.879425	29.596008	1
Ta-tsien-lou (Kangding), Sichuan	B_thaidina	101.958140	29.997335	1
Upper Wasi Gou, Kangding, Sichuan	B_thaidina	102.142502	30.066043	1

Hongxi, Meigu, Sichuan	B_thaidina	103.120473	28.685130	1
Longxi-Hongkou Nature Reserve, Dujiangyan, Sichuan	B_thaidina	103.663391	31.097091	1
Chiufunshan, Szechuan	B_thaidina	103.864035	31.372552	1
Pengzhou, Sichuan	B_thaidina	103.897900	31.299516	1
Qingcheng Shan, Dujiangyan, Sichuan	B_thaidina	103.521152	30.982234	1
Longxigou, Jiulong, Sichuan	B_thaidina	101.542591	28.851659	1
20 km S. of Jiulong, Sichuan	B_thaidina	101.648462	28.816427	1
Lizi Ping, Muli, Sichuan	B_thaidina	101.214995	28.003555	1
Heizhu Gou, Ebian, Sichuan	B_thaidina	103.092561	29.012637	1
Headwater of Jialing River, Tsinling, Shaanxi	B_thaidina	106.935947	34.247600	1
Tapaishan im Tsinling, Sued-Shensi	B_thaidina	107.814101	33.960308	1
Diaoyutai, Taibai Shan, Shaanxi	B_thaidina	107.476877	34.165352	1
Laoxiancheng, Taibai Shan, Shaanxi	B_thaidina	107.757567	33.794706	1
Hougou, Taibai Shan, Shaanxi	B_thaidina	107.370756	34.063692	1
Hualong Shan Nature Reserve, Shaanxi	B_thaidina	109.175944	32.033930	1
Maiji, Xiaolong Shan, Gansu	B_thaidina	105.978537	34.403990	1
Maiji Shan, Gansu	B_thaidina	106.342150	34.285779	1
Yunping, Xiaolong Shan, Gansu	B_thaidina	106.504828	33.594281	1
near Shangdao Yu, Gansu	B_thaidina	104.157040	33.674818	1
near Diebu, Gansu	B_thaidina	103.112819	34.193928	1

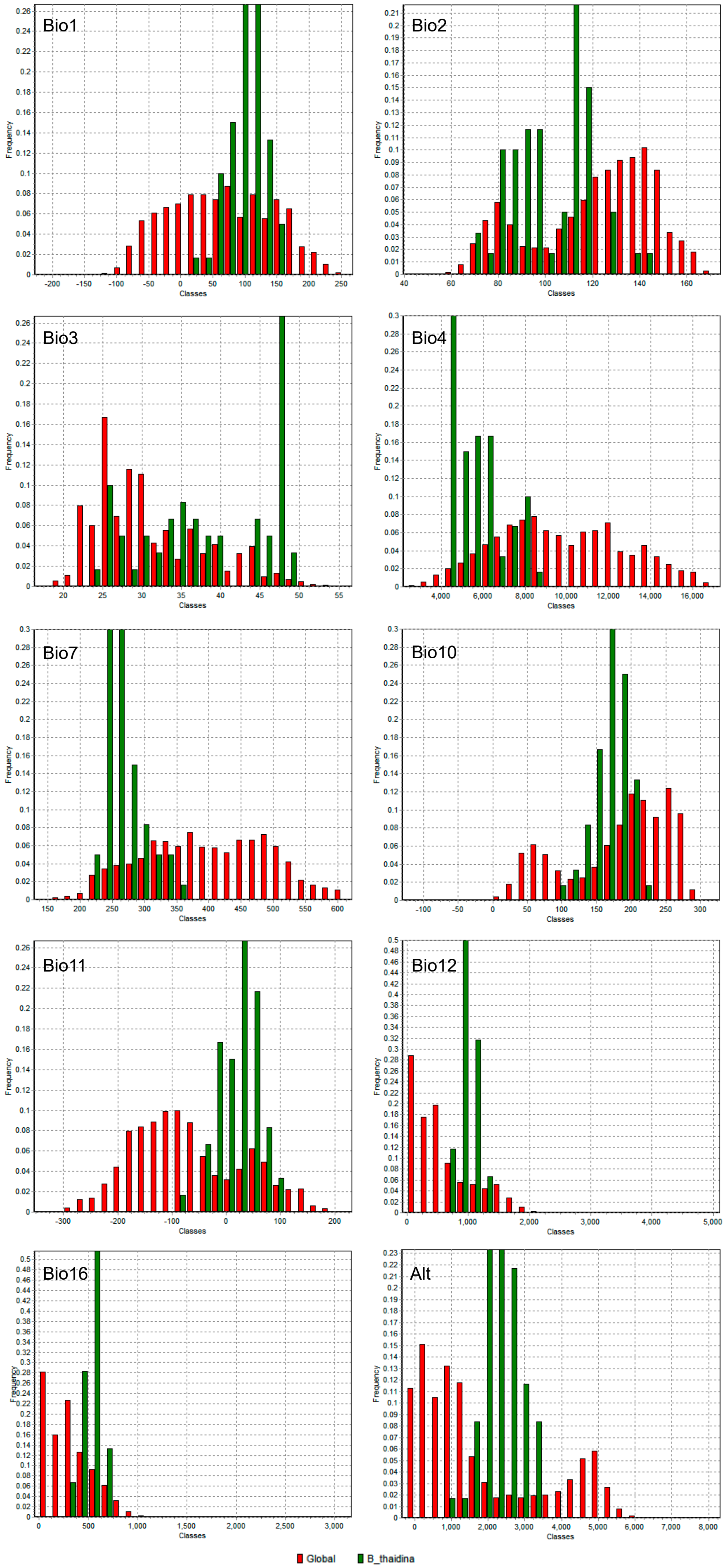

Figure S1. Frequency distribution of EGVs in China and the range of *B. thaidina*
